# Absent Audiovisual Integration Elicited by Peripheral Stimuli in Parkinson's Disease

**DOI:** 10.1155/2018/1648017

**Published:** 2018-04-12

**Authors:** Yanna Ren, Keisuke Suzuki, Weiping Yang, Yanling Ren, Fengxia Wu, Jiajia Yang, Satoshi Takahashi, Yoshimichi Ejima, Jinglong Wu, Koichi Hirata

**Affiliations:** ^1^Department of Psychology, Medical Humanities College, Guiyang University of Chinese Medicine, Guiyang 550025, China; ^2^Cognitive Neuroscience Laboratory, Graduate School of Natural Science and Technology, Okayama University, Okayama 7008530, Japan; ^3^Department of Neurology, Dokkyo Medical University, Tochigi 3210293, Japan; ^4^Department of Psychology, Hubei University, Wuhan 430062, China; ^5^Department of Light and Chemical Engineering, Guizhou Light Industry Technical College, Guiyang 550025, China; ^6^Intelligent Robotics Institute, Beijing Institute of Technology, Beijing 100081, China; ^7^Shenzhen Institute of Neuroscience, Shenzhen 518057, China

## Abstract

The basal ganglia, which have been shown to be a significant multisensory hub, are disordered in Parkinson's disease (PD). This study was to investigate the audiovisual integration of peripheral stimuli in PD patients with/without sleep disturbances. Thirty-six age-matched normal controls (NC) and 30 PD patients were recruited for an auditory/visual discrimination experiment. The mean response times for each participant were analyzed using repeated measures ANOVA and race model. The results showed that the response to all stimuli was significantly delayed for PD compared to NC (all *p* < 0.01). The response to audiovisual stimuli was significantly faster than that to unimodal stimuli in both NC and PD (*p* < 0.001). Additionally, audiovisual integration was absent in PD; however, it did occur in NC. Further analysis showed that there was no significant audiovisual integration in PD with/without cognitive impairment or in PD with/without sleep disturbances. Furthermore, audiovisual facilitation was not associated with Hoehn and Yahr stage, disease duration, or the presence of sleep disturbances (all *p* > 0.05). The current results showed that audiovisual multisensory integration for peripheral stimuli is absent in PD regardless of sleep disturbances and further suggested the abnormal audiovisual integration might be a potential early manifestation of PD.

## 1. Introduction

Parkinson's disease (PD) is traditionally recognized as a movement disorder and is characterized by bradykinesia, resting tremor, and rigidity. However, recent evidence suggests that patients with PD also have various nonmotor disturbances such as depression, cognitive impairment, sleep disorders, and olfactory disturbance [1, 2]. Moreover, nonmotor symptoms are sometimes evident prior to the onset of motor symptoms during the “premotor stage” and influence the patients' quality of life. Therefore, many studies have focused on the early detection and management of nonmotor symptoms associated with PD [1].

People obtain dynamic effective information from the complex environment through multiple senses. A number of studies have reported that people with PD present sensory and perceptual impairments [3], including delayed responses to auditory or visual stimuli compared with age-matched healthy controls [4–7]. However, a simple reaction time task includes many steps: identifying and evaluating the stimulus, selecting the appropriate response, and programming and executing the movement. Thus, delay at any one or all of these stages may lead to a delayed reaction time [5]. For PD patients, the observed increase in simple reaction time may result from slowness of movement compared with age-matched normal controls. To quantitatively assess sensory processing speed, it is necessary to remove the redundant time for executing the movement. Of note, individuals are often inundated with stimuli from various sensory modalities, and merging multisensory information is therefore often crucial in making a rapid and accurate response [8–12]. Therefore, the quantitative assessment of multisensory integration processing ability is very meaningful in the investigation of PD.

By comparing reaction times to multisensory stimuli and to individual component unisensory stimuli, quantitative assessment of multisensory integration can be performed while controlling for response time for executing movement in PD [13–15]. A substantial loss of dopaminergic neurons in the basal ganglia is observed in PD [16–18]. Using an extracellular single-cell recording technique, Nagy et al. confirmed that like the superior colliculus and related structures, the basal ganglia (e.g., the caudate nucleus and the substantia nigra) have the ability to integrate multisensory information [18]. The latest studies conducted by Noy et al. showed that individuals might optimally integrate audiovisual cues to synchronize steps during step-by-step walking [19], which indicated the importance of cross-modal integration of visual and auditory signals for PD patients. However, currently few studies have reported the audiovisual integration ability of PD patients. Fearon et al. [13] recently demonstrated that abnormal audiovisual processing occurs in PD patients compared with age-matched healthy controls; in their study, the visual stimuli were presented centrally. Previous studies have shown that the location of the presented stimuli may greatly affect detection and discrimination of the stimuli. The more peripherally a visual or auditory stimulus was located (from 0° to 60°), the slower both the response accuracy and the speed of the response were [20, 21]. Nidiffer et al. [21] found a similar response pattern to audiovisual stimuli. Furthermore, these authors also reported increasing performance facilitation as the stimuli were positioned at more peripheral locations, indicating that the location of the stimulus (central or peripheral) greatly influenced audiovisual integration [21]. However, it is still unknown whether the multisensory integration of peripheral stimuli is altered in the same way as the multisensory integration of centrally located stimuli.

Sleep disturbances that have a detrimental effect on health-related quality of life are a common disabling nonmotor symptom of PD. Such sleep disturbances can occur at any point during the course of PD and even at the initiative stage [22]. Sleep disturbances are estimated to occur in 60–98% of patients with PD [22–24]. Multiple factors contribute to the occurrence of sleep disturbances, including PD-related pathological changes, nocturnal motor/nonmotor symptoms, medication use, and comorbid primary sleep disorders [25]. However, because no investigation has focused on the audiovisual integration of PD patients with sleep disturbances, it is completely unknown whether or not sleep disturbances worsen the abnormal audiovisual integration that occurs in PD.

Using an auditory/visual stimulus discrimination task [26, 27], the present study aimed to quantitatively measure the ability of PD patients to integrate peripheral audiovisual stimuli and to evaluate the effect of sleep disturbances on PD-related peripheral audiovisual integration.

## 2. Methods

### 2.1. Subjects

A total of 36 age-matched healthy volunteers (62–78 years; mean age ± SD, 69.69 ± 4.41) and 30 PD patients (53–82 years; mean age ± SD, 67.53 ± 9.1) participated in this study and finished the experiment successfully. All of the age-matched healthy volunteers who made up the normal control group were randomly recruited from the Okayama Silver Human Resources Center, and all of the PD patients were recruited from outpatient clinics of the Department of Neurology, Dokkyo Medical University Hospital. All the recruited age-matched healthy volunteers agreed to participate in the experiment, and finished the experiment successfully. However, two of the PD patients in the outpatient clinics had no time to attend the experiment. A diagnosis of PD was made according to the established criteria by board-certified neurologists [28], and disease duration of the PD patients was recorded in Table 1. The participants were naïve to the device and to the task. All the participants provided written informed consent to the procedure, which was previously approved by the ethics committee of Okayama University (NC) or of Dokkyo Medical University Hospital (PD) specifically according to the location at which the examination was performed. Additionally, all of the participants in the NC group were in good physical condition and not taking any medications that might have central effects.

### 2.2. Stimuli

**Figure 1:**
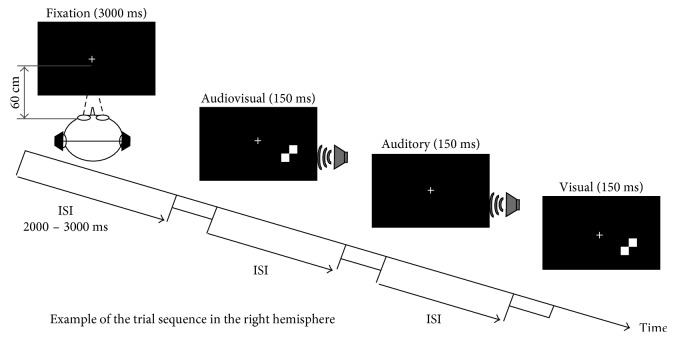
Schematic description of the experimental design. An example of a stimulus used to form a possible trial sequence is shown. After fixation for 3000 ms at the beginning of the session, all the auditory, visual, and audiovisual stimuli were presented randomly with a random interstimulus interval (ISI) of 2000–3000 ms. Following the presentation of each stimulus, the subject was instructed to identify which hemispace was presented by pressing the right or left button of a mouse as rapidly and accurately as possible.

The visual stimulus was a black and white checkerboard image (52 × 52 mm), which was presented on a black background on a 21-inch computer monitor positioned 60 cm in front of the participant's eyes (Figure 1). All visual stimuli (V) were presented on the lower left or lower right quadrant of the screen for 150 ms (at a 12-degree visual angle to the left or right of the center and at a 5-degree angle below the central fixation point). The auditory stimulus was a 1000 Hz sinusoidal tone that was presented randomly to the left or right ear through earphones at approximately 60 dB SPL for 150 ms (10 ms of rise/fall cosine gate). The audiovisual stimuli (AV) were presented through a combination of the visual and auditory stimuli. Each subject participated in only one session consisting of 50 visual stimuli, 50 auditory stimuli, and 50 audiovisual stimuli.

### 2.3. Experimental Procedure

Each subject was instructed on how to complete the experiment, which consisted of two parts, a questionnaire assessment session and an auditory/visual stimuli discrimination session. All of the questionnaires were completed in the outpatient department or in a special quiet room with the assistance of professional staff. For the auditory/visual discrimination session, each subject was tested individually in a quiet room free from external noise and distraction, thereby minimizing the possible influence of any physiological effects.

#### 2.3.1. Questionnaire Assessments

Each participant's overall cognitive function was estimated using the Montreal Cognitive Assessment (MOCA) [29]. The Japanese versions of the Parkinson's Disease Sleep Scale-2 (PDSS-2) [30], the Epworth Sleepiness Scale (ESS) [31, 32], and the Pittsburgh Sleep Quality Index (PSQI) [33] were used to evaluate sleep conditions. Additionally, for the PD patients, Hoehn and Yahr (HY) staging was used to rate disease severity [34], and the duration of the disease and Levodopa equivalent dose (LED) used were obtained from the attending physician after receiving the patient's permission. This session lasted approximately 40 mins.

#### 2.3.2. Auditory/Visual Stimuli Discrimination Session

The subjects were instructed to perform an auditory/visual stimuli discrimination task in a quiet room (a laboratory room at Okayama University or at Dokkyo Medical University Hospital, Japan) with their eyes fixed on the fixation cross (Figure 1). All of the participants were naïve to the device and to the task. Stimulus presentation and response collection were conducted using Presentation software (Neurobehavioral Systems Inc., Albany, California, USA). At the beginning of the task, the subjects were presented with a fixation cross for 3000 ms. Following fixation, all stimuli were presented randomly, and the subjects were instructed to identify which hemispace the stimulus was presented in. They were instructed to press the appropriate button (the right button if the stimulus was presented in the right hemispace and the left button if the stimulus was presented in the left hemispace) as rapidly and accurately as possible. Each stimulus was followed by an interstimulus interval (ISI) that varied randomly in duration from 2000 to 3000 ms for subject response and rest. This session lasted approximately 6.3 mins.

### 2.4. Data Analysis

#### 2.4.1. Questionnaire Assessments

The subject was defined as a poor sleeper if he or she had a global PDSS-2 score ≥15 [30]. The subject was recognized as having excessive daytime sleepiness if he or she had a total ESS score ≥11 [32] and was indicated as having insomnia if the overall PSQI score was ≥6 [35]. In the current study, we divided the PD patients into those with and those without sleep disturbances according to their PDSS-2 scores. The global score was computed separately for each subject in every assessment, and the resulting data were subjected to a one-sample *t*-test (two-tailed).

#### 2.4.2. Mean Response Time and Hit Rates

In the auditory/visual discrimination session, the hit rates and mean response times were computed separately for each subject and in each condition. The hit rate is the percentage of correct responses (the response time falls within the average time period ± 2SD) relative to the total number of stimuli. The mean response time was determined as the average response time for all correct responses. Finally, the data were subjected to 2 groups (NC and PD) ^∗^3 conditions (A, V, and AV) repeated measures ANOVA followed by post hoc tests to analyze the main effect and factors interaction.

#### 2.4.3. Race Model

In the current study, audiovisual integration was evaluated using the redundant nature effect. To examine the redundant nature effect of multisensory integration for the bimodal stimuli condition, we reanalyzed the mean response times using cumulative distribution functions (CDFs). The bimodal AV data were compared with an independent race model, which is a statistical prediction model that uses the CDF of the summed probabilities of the visual and auditory responses [14, 15]. The model permits a direct comparison between the multisensory condition and the predicted probability of unimodal conditions, [*P*(A)+*P*(V)] − [*P*(A) × *P*(V)], by segmenting the subject-specific CDFs for each condition using 10 ms time bins. *P*(A) is the probability of responding within a given time to a unimodal auditory trial, and *P*(V) is the probability of responding within a given time to a unimodal visual trial. If the probability of a response to AV is significantly greater than that predicted by the summed probabilities of A and V, neural audiovisual integration of the two unimodal inputs is considered to be occurred [14, 15]. Then, the redundant nature effect of multisensory conditions was defined by subtracting a subject's race model from his or her audiovisual CDFs at each time bin to generate a difference curve for each subject. A one-sample *t*-test (two-tailed) was then performed for each time bin within each of the groups (the NC and PD groups) to identify significant deviations (*p* < 0.05) by comparing the value at each time bin with zero.

#### 2.4.4. Correlation Analysis

For PD patients, there was no significant audiovisual integration; therefore, the bimodal response facilitation was assessed through the interactive index (ii) [36]. Correlation analysis was conducted to examine the relationship between the degree of response facilitation and H&Y stage or disease duration. The variables AV, V, and A represent the mean response times to each stimulus. All of the data were subjected to bivariate correlations analysis (*Pearson's correlation, two-tailed*):

(1) $$\begin{array}{c} \text{ii}=\frac{\max\left(\text{A;V}\right)-\mathrm{AV}}{\max\left(\text{A;V}\right)}\times 100\%. \end{array}$$

## 3. Results

### 3.1. Questionnaire Assessment

A one-sample *t*-test (two-tailed) was conducted between the NC and PD groups for Age, Education, MOCA score, PDSS-2 score, ESS score, and PSQI score, respectively. No significant difference was found between the two groups for Age (*p*=0.21), Education (*p*=0.4), or PSQI score (*p*=0.68). However, there was a significant difference for MOCA score (*p* < 0.001), indicating the presence of cognitive impairment in PD. Furthermore, significant differences were also found for PDSS-2 score (*p*=0.02) and ESS score (*p*=0.04), showing that obvious sleep disturbances occurred in some PD patients (Table 2).

### 3.2. Mean Response Time and Hit Rates

**Figure 2:**
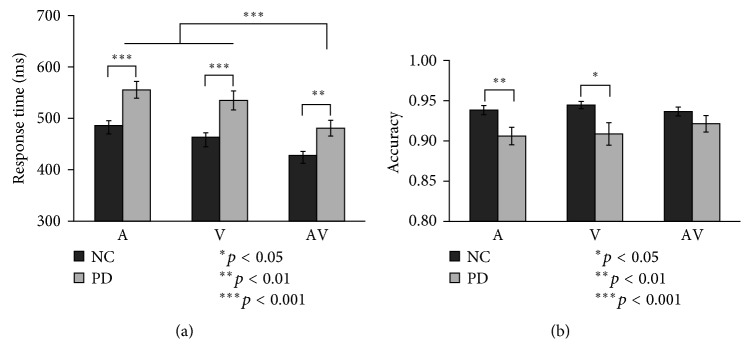
Average reaction times and hit rates by modality and group (error bars indicate the SEM) (A: auditory-only stimulus; V: visual-only stimulus; AV: audiovisual stimulus; ^∗^*p* < 0.05).

The mean response times and hit rates for NC and PD patients are shown in detail in Table 3. Analysis using 3 (modality) ^∗^ 2 (hemispace) repeated measures ANOVA showed main effects of modality in both the NC group [*F*(2,68)=43.06, *p* < 0.001] and the PD patients [*F*(2,58)=31.91, *p* < 0.001]; however, no significant main effects of hemispace were found in either the NC group [*F*(1,34)=5.46, *p*=0.062] or the PD patients [*F*(1,29)=1.63, *p*=0.21]. Therefore, the data from the left and right hemispaces were combined. The mean response times and hit rates for NC and PD are presented in Figure 2.

Analysis of the response times using 2 groups (NC and PD) ^∗^ 3 modalities (A,V, AV) repeated measures ANOVA showed a significant main group effect [*F*(1,63)=14.75, *p* < 0.001]. The pairwise comparisons showed that for the NC group, the responses to bimodal AV stimuli were significantly faster than the responses to unimodal visual (*p* < 0.001) or auditory (*p* < 0.001) stimuli. However, there was no significant difference in the response of the NC group to unimodal visual and auditory stimuli (*p*=0.56). For the PD group, the responses to bimodal AV stimuli were significantly faster than the responses to unimodal visual (*p* < 0.001) or auditory (*p* < 0.001) stimuli. However, there was no significant difference in the response to unimodal visual and auditory stimuli (*p*=0.13). Additionally, there was a significant main effect of modality [*F*(2,126)=72.78, *p* < 0.001]. The pairwise comparisons between the NC and PD groups showed significantly faster responses for NC than those for PD to unimodal visual (*p* < 0.001), auditory (*p* < 0.001), and audiovisual stimuli (*p*=0.002). However, there was no significant interaction between group and modality [*F*(2,126)=1.62, *p*=0.21].

Analysis of the hit rates using 2 Group (NC and PD) ^∗^3 Modality (A,V,AV) repeated measures ANOVA showed a significant main effect of group [*F*(2,63)=8.68, *p*=0.005], with a higher accuracy for the NC group than for the PD group. The pairwise comparisons between the NC and PD groups showed significantly higher accuracy for NC than for PD to unimodal visual (*p*=0.012) and auditory (*p*=0.008) stimuli; however, no significant difference was found for the bimodal AV condition. However, there was no main effect of modality [*F*(2,126)=0.52, *p*=0.59] and no interaction between group and modality [*F*(2,126)=1.31, *p*=0.27].

### 3.3. Race Model Comparisons

**Figure 3:**
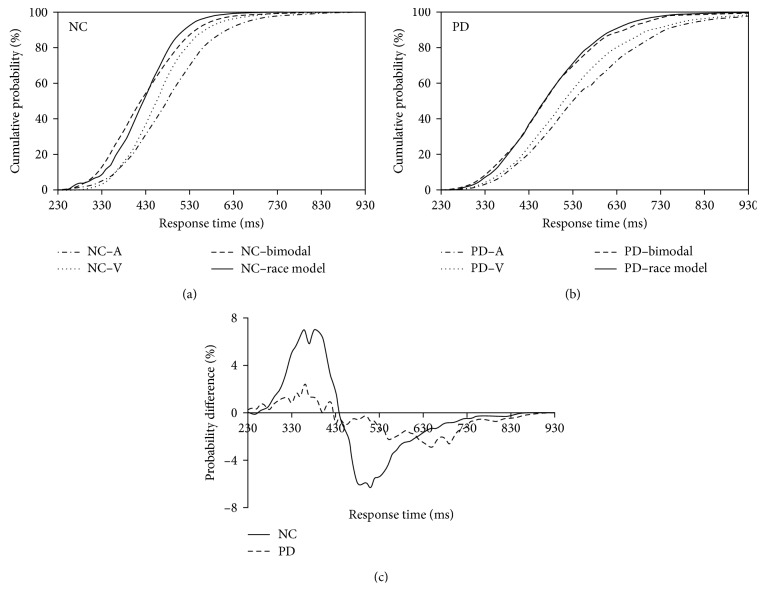
Distributions of response times. (a) Cumulative distribution functions (CDFs) for the discrimination response times to auditory, visual, and audiovisual stimuli and for the race model for NC. (b) CDFs for PD. (c) Significant audiovisual integration was found in NC (solid line), but not in PD patients (dotted line).

A race model was used to analyze the RTs to evaluate the redundant nature effect of the multisensory stimuli under each of the experimental conditions (Figures 3(a) and 3(b)). The relationship was compared by subtracting the race model CDFs from the bimodal AV CDFs for each group (Figure 3(c)). Significant audiovisual interactions (*p* < 0.05, one-sample *t*-test) were found for the NC group (Figure 3(c), solid line); however, there were no significant differences between the responses to bimodal stimuli and the predicted race model for the PD patients (Figure 3(c), dotted line) (*p* > 0.05).

**Figure 4:**
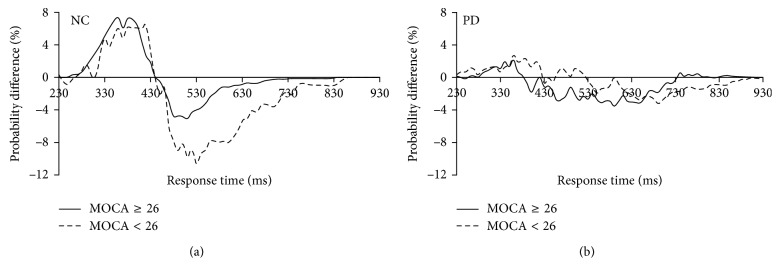
Direct comparison of audiovisual performance relative to the predicted race model. Significant audiovisual integration was found in both NC with (dotted line) and without (solid line) cognitive impairment (a). No significant audiovisual integration was occurred with (dotted line) or without (solid line) cognitive impairment (b).

**Figure 5:**
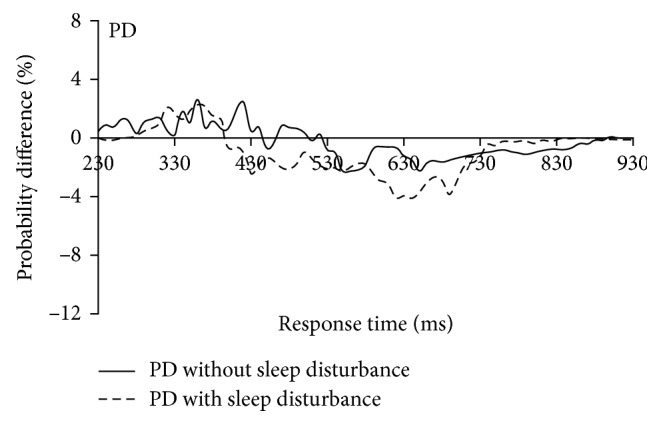
Direct comparison of audiovisual performance relative to the predicted race model. No significant audiovisual integration was found in both PD with sleep disturbance group (dotted line) and without sleep disturbance group (solid line).

The foregoing results indicate that there was significant diversity in audiovisual interaction between the PD patients and the age-matched NC group. To further examine the possible cause of the significant cognitive functional difference between these two groups, we divided each group into two subgroups based on the individuals' MOCA scores (cutoff = 26). A similar race model analysis method was used for the further analyses. The results indicated that there was no significant difference between the NC subgroups (*p* > 0.05; Figure 4(a)) or between the PD subgroups (*p* > 0.05; Figure 4(b)). Additionally, we further divided PD patients into PD patients with sleep disturbance and PD patients without sleep disturbance based on their PDSS-2 scores (Table 1). The redundant nature analysis results showed no significant audiovisual integration for either the PD with sleep disturbance or the PD without sleep disturbance groups (Figure 5). Furthermore, the one-sample *t*-test (two-tailed) found that there was no significant difference between PD patients with sleep disturbance and PD patients without sleep disturbance (*p* > 0.05).

### 3.4. Correlation Analysis

**Figure 6:**
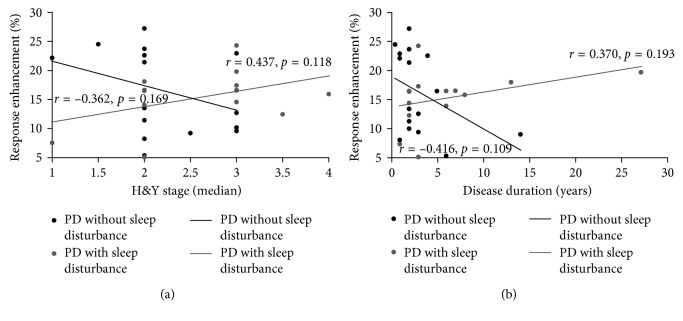
Correlation of H&Y stage and disease duration with response facilitation. (a) No significant relationship between H&Y stage and response facilitation. *x*-axis: H&Y stage; *y*-axis: response facilitation. (b) No significant relationship between disease duration and response facilitation. *x*-axis: disease duration; *y*-axis: response facilitation.

Figure 6 shows the results of the correlation analysis. The results indicate that there was no significant relationship between H&Y stage and response facilitation either for the PD with sleep disturbance group (*r*=0.437, *p*=0.118) or for the PD without sleep disturbance group (*r*=−0.362, *p*=0.169), nor was there a significant relationship between disease duration and response facilitation for either the PD with sleep disturbance group (*r*=0.370, *p*=0.193) or the PD without sleep disturbance group (*r*=−0.416, *p*=0.109).

## 4. Discussion

In this study, we examined the audiovisual multisensory integration elicited by peripheral stimuli in PD. The results showed that no significant audiovisual integration occurred in PD regardless of the presence of cognitive deficits or sleep disturbance; however, such integration did occur in age-matched normal controls. In addition, there were no significant correlations between audiovisual facilitation and H&Y stage or disease duration.

### 4.1. Absent Audiovisual Integration in PD

Regarding the investigation of multisensory integration in PD, this is the first study to quantitatively examine audiovisual multisensory integration to stimuli presented peripherally to PD patients and to report that no significant audiovisual integration was observed. Consistent with the results of a previous study, audiovisual processing in PD was found to be abnormal [13]. To some degree, the current results lead to a conclusion similar to that expressed by Fearon et al. They found that audiovisual processing in PD was attenuated but did exist and further reported that it was correlated with freezing of gait and disease duration [13]. Investigation of visual processing by fMRI showed that in PD patients, the neural response of the visual cortex to peripheral stimuli was weaker than the response to central stimuli [37]. Consistent with the inverse effectiveness principle of multisensory integration, audiovisual integration was greater when the stimuli were positioned at more peripheral locations (0° versus 30° and 60°) [21]. Therefore, the partial differences between our results and those of the previous study might be associated with the visual angle at which the stimuli were presented. To date, although there is no evidence of pathology in the visual cortex of PD patients, the present studies have implied the dysfunction of visual perception, and reduced activity in visual association cortices and primary visual cortex was found in PD [38, 39]. The regions traditionally considered to be sensory-specific (e.g., the primary visual cortex) have been proven an audiovisual integration effect as compensatory phenomena [40–43]. Therefore, it is reasonable for the abnormal audiovisual integration in PD patients.

Besides, recent studies have provided evidence that the subcortical basal ganglia have multisensory properties [17, 18]. Nagy et al. reported that the caudate nucleus and substantia nigra of anesthetized cats can respond to visual, auditory, or somatosensory stimulation alone as well to multisensory stimuli. In addition, the multisensory units of the caudate nucleus and substantia nigra showed significant cross-modal interaction, displaying additive or super-additive response facilitation to multisensory simulation. There is known to be a basal ganglia disorder in PD, as evidenced by cell death in the substantia nigra and the ventral (front) part of the pars compacta as well as substantial loss of dopaminergic neurons in the substantia nigra [24, 44, 45]. The higher audiovisual integration of peripheral stimuli might be susceptible to basal ganglia impairment; thus, it seems very possible that disorders of the basal ganglia may lead to the absence of audiovisual integration processing.

Additionally, attention can also greatly modulate audiovisual integration, and audiovisual integration has been shown to be greater in attended tasks than in unattended tasks [46–50]. Numerous behavioral and electroencephalographic studies have provided evidence for the existence of attentional deficits, for example, visual attention and auditory attention, in PD [49–54]. Therefore, the decrease in attention that occurs in PD might also contribute to the absence of audiovisual integration.

### 4.2. Absent Audiovisual Integration Independent of Cognitive Deficits and Sleep Disturbance in PD

In the present study, we examined 30 clinical PD patients who successfully completed the auditory/visual stimuli discrimination experiment independently without serious cognitive deficits or motor impairment. The demographic information showed lower MOCA scores for the PD patients than for their age-matched normal controls, and a further *t*-test (two-tailed) analysis revealed significant differences between the two groups. Our previous study showed decreased audiovisual integration both in divided attention and selective attention tasks for patients with mild cognitive impairment, and audiovisual integration further decreased significantly in Alzheimer's disease patients [55]. These results suggested that the cognitive deficits that occur in PD might lead to abnormal audiovisual integration. According to their MOCA scores, we divided the PD patients into two groups (cutoff = 26). The analysis revealed that there was no significant difference in audiovisual integration between the two sub-PD groups (MOCA scores of ≥26 and <26). According to the results, we propose that although cognitive deficits attenuate audiovisual integration, as found in our previous study, basal ganglia disorders might be the most critical factor associated with the lack of audiovisual integration in PD patients. In our study, we also divided the normal control group into two subgroups according to their MOCA scores. In that group, there were only three participants with MOCA scores below 26 (two participants with scores of 25 and one with a score of 24), and these participants showed no significant cognitive impairment. Although the data for normal control participants with MOCA scores below 26 are insufficient for statistical analysis, the average probability difference curve of these participants is similar to that of the group with MOCA scores of 26 or greater. This result further suggests that audiovisual multisensory integration is much more sensitive to the basal ganglia disorder that occurs in PD than to slight alterations in cognitive function.

Additionally, in the present study, we examined the audiovisual integration of peripheral stimuli in PD patients with or without sleep disturbance. According to the PDSS-2 scores, 14 PD patients (47%) had sleep disturbances; based on these scores, we divided all the participants into two groups: those with and those without sleep disturbances. However, we found no significant difference in multisensory audiovisual integration in the two subgroups. These results further suggest that audiovisual integration of peripheral stimuli might be especially sensitive to basal ganglia disorders and that sleep disturbance was not the key factor determining the lack of audiovisual integration in these patients.

To further investigate whether the ability to integrate peripheral audiovisual stimuli is reserved in the early stage of PD and disappears with the progression of the disease in the current study, we evaluated the correlation of H&Y stage and disease duration with audiovisual multisensory integration through relative unisensory response time facilitation. No significant relationship was found between the bimodal response facilitation and H&Y stage or disease duration (all *p* > 0.05), regardless of the presence of sleep disturbance. The results confirm that the abnormal audiovisual multisensory processing occurred at an early PD stage. Together with the results of the previous study, these results further suggest that deficiencies in audiovisual multisensory processing might be a potential early manifestation of PD [13].

### 4.3. Limitations

The main limitation of the current study is the systemic alteration of audiovisual integration that occurred as a function of the change in the visual angle at which the stimuli are presented. The lack of objective assessments for the sleep status, such as polysomnography or actigraphy, and the limited battery (MOCA) used in this study could have influenced the results. The multiple comparisons employed in this study were not corrected because our study was exploratory in nature, which may have included some significant findings by chance. Additionally, as the sample was relatively small, we were unable to divide the PD patients according to motor symptoms and Levodopa equivalent dose in detail. Furthermore, EEG investigation is needed to evaluate the time course of audiovisual integration in PD to further verify whether the observed lack of audiovisual integration of peripheral stimuli occurs at the early stage of audiovisual integration.

## 5. Conclusions

The current study provides the first evidence that the audiovisual multisensory integration of peripheral stimuli is absent in PD patients regardless of the presence of sleep disturbances. The results of the study further suggest that abnormal audiovisual integration may be a potential early manifestation of PD.

## Figures and Tables

**Table 1:** Demographics of Parkinson's disease patients with/without sleep disturbance according to PDSS-2, ESS, and PSQI scores.

	All PD patients	PD with sleep disturbances	PD without sleep disturbances
*N*	30	14	16
Age (y)	67.5 (1.6)	69.6 (2.3)	65.7 (0.2)
Gender (F : M)	17 : 13	7 : 7	10 : 6
H&Y stage	2.4 (0.1)	2.6 (0.2)	2.3 (0.2)
Disease duration (y)	4.5 (1.0)	5.8 (1.9)	3.4 (0.8)
Education (y)	12.4 (0.4)	12.1 (0.7)	12.6 (0.6)
PDSS-2^∗∗∗^	15.2 (2.1)	24 (3.2)	7.6 (0.8)
ESS	7.8 (1.0)	8.5 (1.6)	7.2 (1.5)
PSQI	6.9 (1.2)	8.9 (2.2)	5.1 (1.1)
MOCA	24.2 (0.5)	24.3 (0.7)	24.1 (0.8)
LED (mg/day)	452 (67)	508 (90)	403 (100)

Data are presented as the mean  ±  standard error of the mean (SEM). F: female; M: male; H&Y stage: Hoehn & Yahr staging system (stages from 1 to 5; a higher score reflects more severe symptoms); PDSS-2: Parkinson's Disease Sleep Scale (score greater than the cutoff of 15 indicates a poor sleeper); ESS: Epworth Sleepiness Scale (score greater than the cutoff of 11 indicates excessive daytime sleepiness); PSQI: Pittsburgh Sleep Quality Index (score greater than the cutoff of 6 suggests possible insomnia); MOCA: Montreal Cognitive Assessment (score greater than the cutoff of 26 reflects normal cognition). ^∗∗∗^*p* < 0.001 indicates a statistically significant difference between the PD group with sleep disturbances and the PD group without sleep disturbances.

**Table 2:** Comparisons of MOCA, PDSS-2, and ESS scores between NC and PD groups.

	Simple size, number	Female/male	Age (y)	Education (y)	MOCA^∗∗∗^	PDSS-2^∗^	ESS^∗^	PSQI
NC	36	18/18	69.69 ± 0.74	12.86 ± 0.34	26.78 ± 0.33	10.03 ± 0.97	5.28 ± 0.65	6.33 ± 0.75
PD	30	16/14	67.53 ± 1.63	12.40 ± 0.44	24.17 ± 0.53	15.27 ± 2.10	7.80 ± 1.08	6.90 ± 1.20

Data are presented as the mean ± standard error of the mean (SEM). NC: age-matched normal controls; PD: Parkinson's disease patients. ^∗^*p* < 0.05, ^∗∗∗^*p* < 0.001; statistically significant difference between the NC group and the PD group.

**Table 3:** The mean response times and hit rates for NC and PD patients.

	NC		PD	
	Right	Left	Right	Left
Response time (ms)				
Auditory	494.6 (8.8)	475.7 (10.9)	576.3 (18.7)	537.1 (17.9)
Visual	467.8 (9.1)	459.2 (8.4)	543.4 (18.8)	527.1 (21.4)
Audiovisual	431.2 (7.7)	425.7 (9.4)	482.0 (15.1)	481.7 (17.9)

Hit rates				
Auditory	0.95 (0.006)	0.95 (0.007)	0.91 (0.01)	0.91 (0.02)
Visual	0.95 (0.007)	0.95 (0.005)	0.91 (0.01)	0.91 (0.02)
Audiovisual	0.95 (0.005)	0.95 (0.007)	0.92 (0.02)	0.92 (0.01)

Data are presented as the mean ± standard error of the mean (SEM). A: auditory-only stimulus; V: visual-only stimulus; AV: audiovisual stimulus.
